# The activity of cuproptosis pathway calculated by AUCell algorithm was employed to construct cuproptosis landscape in lung adenocarcinoma

**DOI:** 10.1007/s12672-023-00755-7

**Published:** 2023-07-23

**Authors:** Weixian Lin, Jiaren Wang, Jing Ge, Rui Zhou, Yahui Hu, Lushan Xiao, Quanzhou Peng, Zemao Zheng

**Affiliations:** 1 Department of Respiratory and Critical Care Medicine, Nanfang Hospital, Southern Medical University, Guangzhou, Guangdong China; 2 The First Clinical Medical School, Southern Medical University, Guangdong Guangzhou, China; 3 Department of Pediatrics, Nanfang Hospital, Southern Medical University, Guangdong Guangzhou, China; 4 Department of Oncology, Nanfang Hospital, Southern Medical University, Guangdong Guangzhou, China; 5 Present Address: Guangdong Province Key Laboratory of Molecular Tumor Pathology, Guangzhou, Guangdong China; 6 Department of Huiqiao Medical Centre, Nanfang Hospital, Southern Medical University, Guangzhou, Guangdong China; 7 Department of Infectious Diseases, Nanfang Hospital, Southern Medical University, Guangdong Guangzhou, China; 8 Department of Pathology, Shenzhen People’s Hospital, The Second Clinical Medical College of Jinan University, The First Affiliated Hospital of Southern University of Science and Technology, Shenzhen, China

**Keywords:** Lung adenocarcinoma, Cuproptosis, Single cell landscape, TLE1, Predictive model

## Abstract

Cuproptosis is a recently described copper-dependent cell death pathway. Consequently, there are still few studies on lung adenocarcinoma (LUAD)-related cuproptosis, and we aimed to deepen in this matter. In this study, data from 503 patients with lung cancer from the TCGA-LUAD cohort data collection and 11 LUAD single-cells from GSE131907 as well as from 10 genes associated with cuproptosis were analyzed. The AUCell R package was used to determine the copper-dependent cell death pathway activity for each cell subpopulation, calculate the CellChat score, and display cell communication for each cell subpopulation. The PROGENy score was calculated to show the scores of tumor-related pathways in different cell populations. GO and KEGG analyses were used to calculate pathway activity. Univariate COX and random forest analyses were used to screen prognosis-associated genes and construct models. The ssGSEA and xCell algorithms were used to calculate the immunocyte infiltration score. Based on data from the GDSC database, the drug sensitivity score was calculated using oncoPredict. Finally, in vitro experiments were performed to determine the role of TLE1, the most important gene in the prognostic model. The 11 LUAD single-cell samples were classified into 8 different cell populations, from which epithelial cells showed the highest copper-dependent cell death pathway activity. Epithelial cell subsets were significantly positively correlated with MAKP, hypoxia, and other pathways. In addition, cell subgroup communication showed highly active collagen and APP pathways. Using the Findmark algorithm, differentially expressed genes (DEGs) between epithelial and other cell types were identified. Combined with the bulk data in the TCGA-LUAD database, DEGs were enriched in pathways such as EGFR tyrosine kinase inhibitor resistance, Hippo signaling pathway, and tight junction. Subsequently, we selected 4 genes (out of 112) with prognostic significance, ANKRD29, RHOV, TLE1, and NPAS2, and used them to construct a prognostic model. The high- and low-risk groups, distinguished by the median risk score, showed significantly different prognoses. Finally, we chose TLE1 as a biomarker based on the relative importance score in the prognostic model. In vitro experiments showed that TLE1 promotes tumor proliferation and migration and inhibits apoptosis.

**Supplementary Information:**

The online version contains supplementary material available at 10.1007/s12672-023-00755-7.

## Background

Lung cancer is commonly diagnosed as lung adenocarcinoma (LUAD) [1]. It has the highest morbidity and mortality rates worldwide and poses a serious threat to human health [1]. Treatment options for lung cancer encompass chemotherapy, radiotherapy, and surgery [2]. However, the insidious nature of lung adenocarcinoma (LUAD) makes early diagnosis challenging. Consequently, most patients are diagnosed in the advanced stages [3]. This late diagnosis significantly restricts the applicability of surgical interventions and exacerbates health complications due to the metastatic spread of cancer cells [4]. To improve the outcome of LUAD, reasonable treatments should be implemented. Currently, tumor risk score prediction signatures are used to determine the survival rate of patients [5, 6]. Therefore, patient prognosis can be accurately predicted by these tools, which are gradually being used in clinical settings [7, 8]. As a result, prognostic features should be urgently identified to predict the long-term survival rates of patients with LUAD.

Cell death is a common part of life; thus, it has always been a focus on life science research. Apoptosis, ferroptosis, pyroptosis, and necroptosis are different types of cell death, and their molecular patterns vary according to the mechanisms used [9–11]. Among them, the classic cell death mechanism described in 2012 has been the subject of recent research: ferroptosis [12, 13]. Significantly, various cellular death subprograms diverge in their initiating triggers, intermediate activation mechanisms, and final biological consequences [13].

It is widely known that heavy metal ions are essential micronutrients for the human body [14–16]; however, it is possible for cells to die if they are exposed to too much or too little metal ions [15]. For example, all life relies on copper, which is typically found at very low levels in mammalian cells, similar to iron ions [16–18]. Increasing the concentration of copper ions in cells above the threshold required for homeostasis can also cause cytotoxicity [18]. Tsvetkov et al. discovered a novel mode of cell death that depends on and is regulated by copper ions; it is called copper-induced cell death or cuproptosis [19]. The mechanism of cuproptosis involves direct binding of copper ions to acylated components of the tricarboxylic acid (TCA) cycle, leading to abnormal aggregation of acylated proteins and loss of iron-sulfur cluster proteins, ultimately leading to proteotoxic stress-mediated cell death [19]. Despite the advances in understanding cuproptosis, its specific role in LUAD remains unclear. There is a gap in knowledge concerning how cuproptosis interacts with other cellular processes and pathways within the context of LUAD, and how these interactions could potentially impact disease progression and patient prognosis. Therefore, the present study was conducted to investigate the involvement of cuproptosis in LUAD, to elucidate its regulatory role, and to explore its potential as a novel therapeutic target for LUAD treatment.

The advantage of using bioinformatics methods in this study lies in their ability to handle large-scale and heterogeneous data [20, 21], thereby enhancing the depth and breadth of our analysis. By integrating gene expression profiles and single-cell data from multiple LUAD samples, we could comprehensively elucidate the complex landscape of cuproptosis in LUAD, providing a resolution unachievable with conventional experimental methods. In this study, we identified seven genes associated with cuproptosis (FDX1, LIAS, LIPT1, DLD, DLAT, PDHA1, and PDHB) and three genes that inhibit it (MTF1, GLS, and CDKN2A). Though it was previously thought that a model of cuproptosis could be constructed based solely on gene expression levels, our application of bioinformatics has revealed that the related subtypes of cuproptosis cannot be fully uncovered using such a method. By calculating the copper-dependent death pathway score and screening for copper-induced death subsets, we were able to identify two types of cuproptosis expression patterns, each associated with different prognoses and tumor microenvironment (TME) characteristics. Importantly, these patterns could potentially serve as a basis for a novel prognostic model for LUAD, thereby providing a possibility for improved patient stratification and personalized treatment strategies.

## Methods

### Data collection

Transcription maps and the corresponding clinical information of patients were extracted from the Cancer Genome Atlas (TCGA)-LUAD database. In addition, 11 lung adenocarcinoma single-cell samples were collected from GES131907 in the Gene Expression Omnibus (GEO) database. No further analysis was conducted on patient data that lacked important clinical or survival parameters. In addition, this study included the following genes associated with cuproptosis: CDKN2A, FDX1, DLD, DLAT, LIAS, GLS, LIPT1, MTF1, PDHA1, and PDHB. We used R 4.1.1 software to postprocess and standardize the original readings of the above data using limma 3.52.2.

### Cell subgroup typing and cell communication identification

Cells were grouped according to the following key cell subset markers: CD79A corresponds to B lymphocytes; RAMP2, VWF, and ACKR1 to endothelial cells; LUM, COL3A1, and DCN to fibroblasts; TPSAB1 and CPA3 to MAST cells; CD8A, CD8B, and CD3D to T lymphocytes; LYZ and C1QB to myeloid cells; S100A2 and SFN to fibroblasts; and NKG7 to NK cells. The AUCell R package was used to determine the activity of the cuproptosis pathway for each cell subset, based on the expression profiles of ten commonly upregulated genes. The AUCell algorithm is an innovative computational method used in the realm of single-cell RNA-Seq data analysis [22]. Its primary goal is to score individual cells based on a predefined set of genes, with an emphasis on gene expression rankings rather than absolute expression levels. This algorithm effectively transforms gene expression matrices into an enrichment score for each cell, essentially providing a measure of the activity of a certain gene set within each individual cell. The methodology behind AUCell involves the calculation of an Area Under the Curve (AUC) for each gene set and cell, thereby creating a robust ranking system that can accommodate various gene expression distributions. This method is highly beneficial for analyzing single-cell data as it allows the identification of cell subsets expressing a specific gene signature, even when the signature is only mildly expressed. The CellChat score was calculated for the eight cell populations in the dimensionality reduction group, and the cell communication of each cell population and the cell communication network within the collagen and APP pathways were displayed. The PROGENy score was calculated to show the scores of tumor-related pathways in different cell populations.

### Functional enrichment analysis

In order to pinpoint genes associated with cuproptosis, the FindMarkers algorithm was deployed for the purpose of discerning differentially expressed genes (DEGs) across distinct cell subpopulations. This identification was based on a stringent criterion of an absolute log fold-change greater than 1 and an adjusted p-value less than 0.05. As the cuproptosis pathway is mainly enriched within epithelial cell subsets, we selected the DEGs among epithelial cell subsets and other cell subgroups for subsequent analysis. ClusterProfiler (version 4.0.5) was used for GO annotation and KEGG pathway enrichment analyses. Statistical significance was set at p ≤ 0.05 and false discovery rate (FDR) ≤ 0.25.

### Construction and verification of a random forest survival model

Univariate COX survival analysis was performed on DEGs using the tinyarray package to identify prognostic genes. Subsequently, a random forest algorithm was employed, which is a robust machine learning method offering a non-parametric and non-linear alternative to traditional Cox regression models [23]. This advanced algorithm is designed to identify intricate interactions between variables, and it has the distinct advantage of being particularly adept at handling high-dimensional genomic data. The selection of this algorithm was guided by its demonstrated superior performance in identifying prognostic markers in cancer and other diseases, as validated by multiple peer-reviewed studies. Leveraging this algorithm, the genes with the most substantial prognostic power were selected and subsequently employed to construct a prognostic model within the TCGA-LUAD cohort. Next, we divided the data into training and validation cohort sets based on a 7:3 ratio and differentiated between high- and low-risk groups using the median of the risk score for subsequent analysis.

### Infiltration of immune cells

We used two algorithms, ssGSEA and xCell, to calculate the immune infiltration score, and visualized them with stacking maps, correlation heat maps, and scatter plots.

### Drug sensitivity assessment

In this study, the R package oncoPredict was used to analyze the sensitivity of each patient with LUAD to chemotherapy drugs using the Cancer Drug Sensitivity Genomic (GDSC) database.

### Cell cultures

Human bronchial epithelial cells HBE135-E6E7 and lung cancer cells A549, NCI-H2073 were purchased from the American Type Culture Collection (ATCC, Rockville, MD, USA) and cultured in KM and DMEM high glucose media supplemented with 10% fetal bovine serum.

### Plasmid construction and transfection

Oligonucleotides encoding shRNA targeting TLE1 and scrambled shRNA were purchased from Aiji Biotechnology (Guangzhou, China). The disordered shRNA was cloned into the pLKO.1 lentiviral vector. A549 cells were transfected with lentivirus alone or carrying the TLE1 overexpression plasmid or the blank vector. Cells were transfected with Lipofectamine 3000 and P3000 reagents, according to the manufacturer’s instructions (Invitrogen, Carlsbad, CA, USA).

### Western blotting

Proteins were extracted from cell samples. Proteins were separated by sodium dodecyl sulfate-polyacrylamide gel electrophoresis (SDS-PAGE) and transferred to polyvinylidene fluoride membranes that were then sealed with 5% milk. The membranes were incubated overnight with a specific primary antibody at 4 °C. For western blotting, ECL Western Blotting Substrate (Solarbio, China) was used after secondary antibody incubation.

### Determination of cell invasiveness

In this migration assay, we used a Boyden chamber (aperture, 8 μm) (BD Biosciences). DMEM containing 15% FBS was placed in the lower chamber, whereas cells were placed in the upper chamber. Cells on the upper side of the filter membrane were removed using a cotton swab, fixed with methanol for 10 min, and stained with 0.05% crystal violet for 24 h. The cells were then observed and counted under a microscope.

### Colony formation assay

The seeded cells were incubated for two weeks in 6-well plates. Paraformaldehyde 4% was used to fix the colonies for 30 min, and then crystal violet (Beyotime, Shanghai, China) was used for two hours to stain them. Statistical analysis was performed after cells were washed twice with fresh PBS and dried at room temperature.

### Apoptosis analysis

Cell apoptosis was analyzed using flow cytometry after pre-cooling with PBS washing and digestion with trypsin digestion solution containing no EDTA (Solarbio, Shanghai, China). Cells were harvested after centrifugation at 1000 rpm for 5 min, followed by 7-AAD (BD Biosciences, number 559, 925, USA) and annexin-APC (BD Biosciences, number 561, 012, USA) staining for 15 min.

### Statistical analysis

All statistical analyses were performed using R software (version 4.1.1). Statistical significance was set at p < 0.05.

## Results

**Fig. 1:**
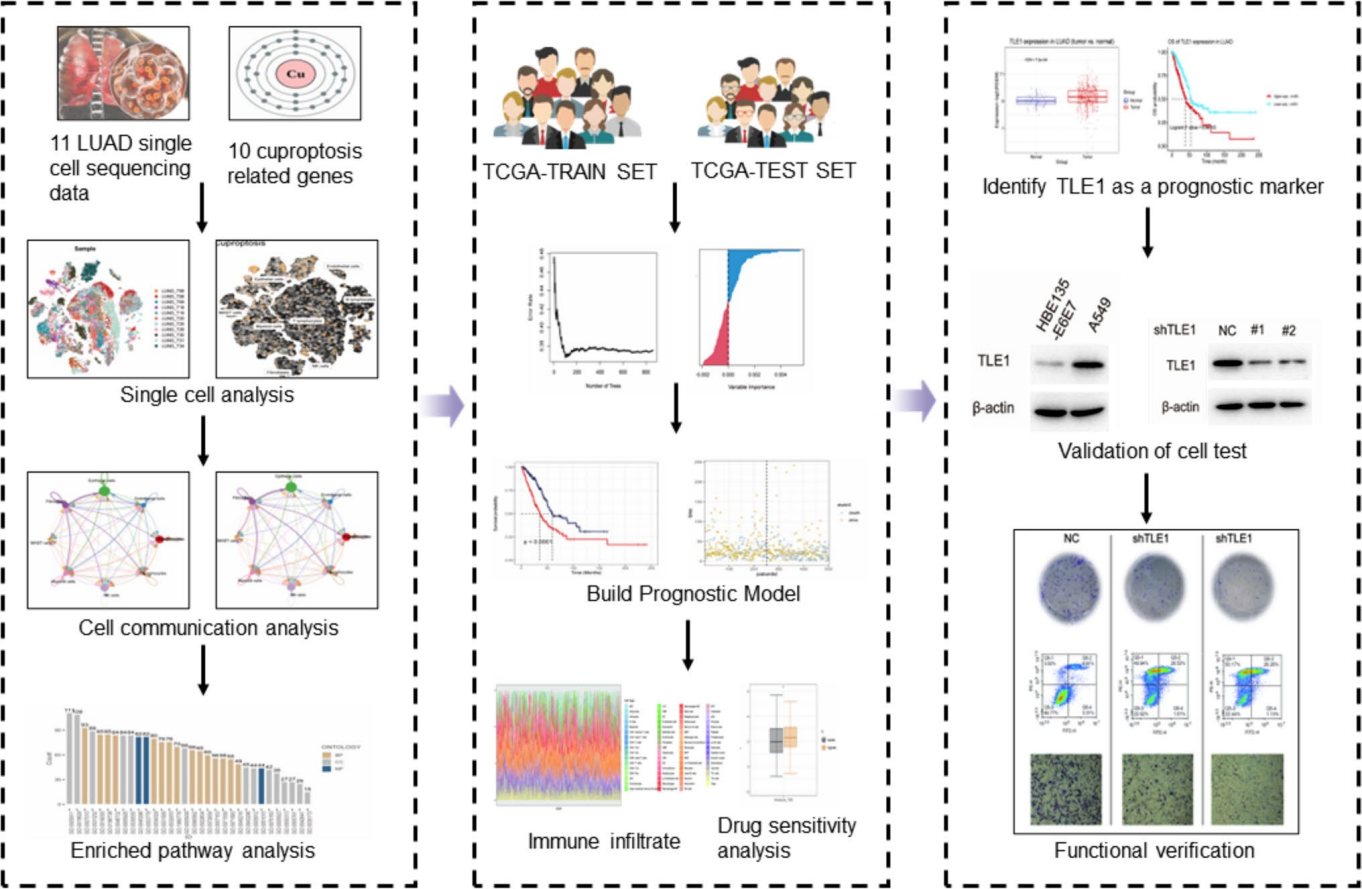
The study flow chart

The flowchart for this study is shown in Fig. 1.

### Transcriptome analysis of LUAD at the single-cell level

**Fig. 2:**
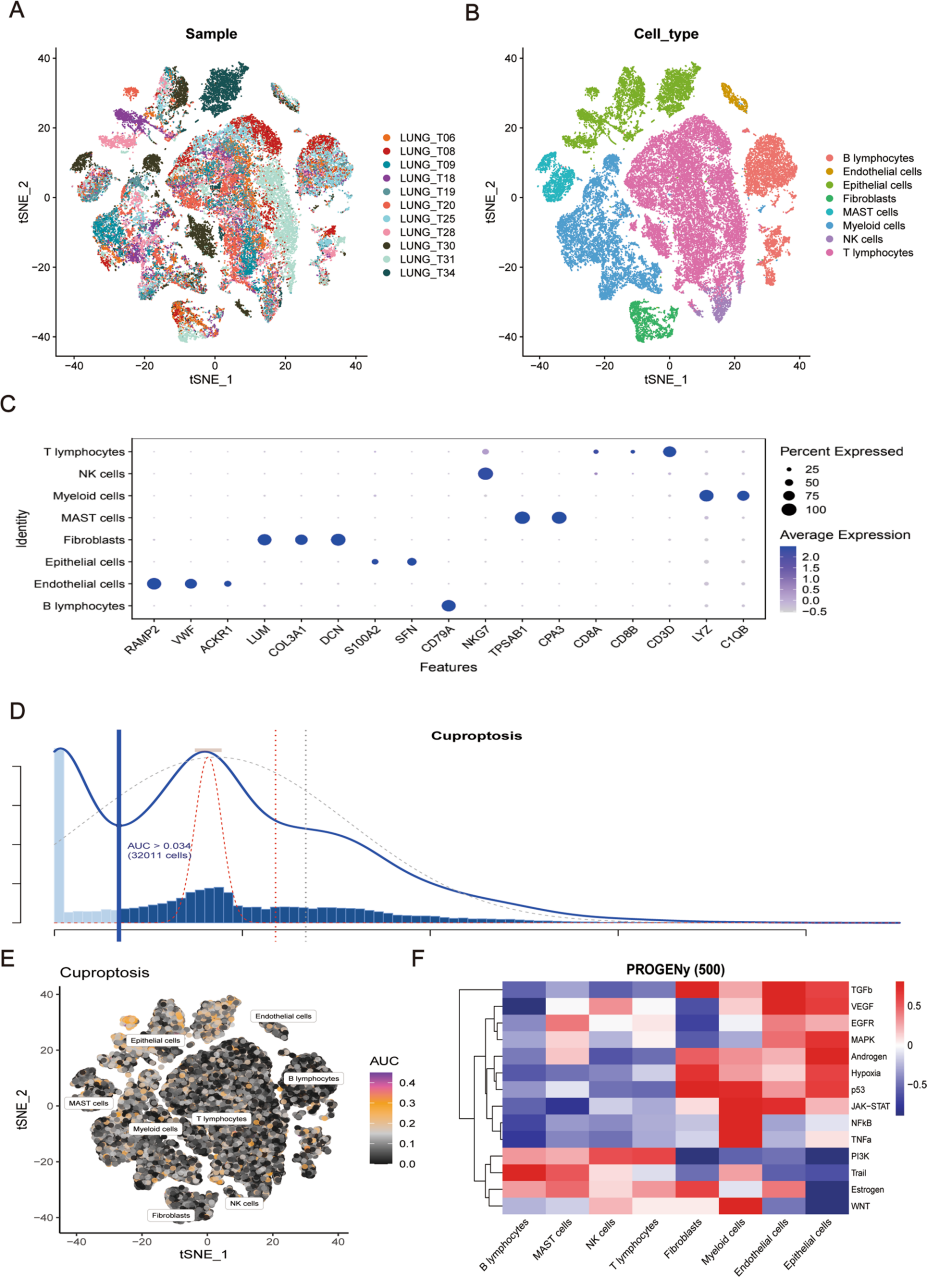
Single-Cell Cluster Analysis of Lung Adenocarcinoma (LUAD) Cell Subsets. **A** t-Distributed Stochastic Neighbor Embedding (t-SNE) plots depicting cluster annotations for LUAD cell subsets. **B** Dimensionality reduction annotated t-SNE plots displaying a total of 8 identified cell subsets. **C** Bubble plots showcasing classic gene expression across the identified cell subsets. **D** Columnar distribution plots displaying the expression of genes related to copper-induced cell death (cuproptosis) across cell subsets. **E** t-SNE plot illustrating the expression of cuproptosis-related genes within each LUAD cell subset. **F** Heatmap depicting the expression of key pathways across different cell subsets

To systematically study the complex intercellular crosstalk of LUAD in multicellular ecosystems, scRNA-seq analysis was performed on 11 LUAD samples after quality control and bimodality removal (Fig. 2A). We then used Seurat to integrate and analyze the sequencing data from the 11 samples. The transcriptomes of eight major cell types were analyzed based on the expression of canonical gene markers (Fig. 2B). Our results showed that cell diversity in patients with LUAD had previously been underestimated. A detailed list of the marker genes analyzed is provided in Fig. 2C.

### Cuproptosis-related pathway score

To evaluate the importance of cuproptosis in each cell subset, we used the AUCell R package to determine the cuproptosis pathway activity in each cell subgroup. The larger the AUC value, the stronger the cuproptosis pathway activity. Figure 2D shows that the number of cells with an AUC value > 0.034 was 32,011. We then constructed a t-SNE diagram to visualize the activity of the cuproptosis pathway in each cell subgroup. In this plot, yellow dots indicate a strong activity, whereas gray dots indicate a weak activity; clearly, the cuproptosis pathway was more active in epithelial cells than in any other group of analyzed cells (Fig. 2E). Consequently, we only used epithelial cells for further analysis.

PROGENy scores are useful for inferring the activity of tumor-associated pathways in different cell populations; we found that epithelial cell subsets were enriched in several pathways, such as TGF-β, MAPK, and hypoxia pathways (Fig. 2F). The above results suggest that LUAD lesions are mainly concentrated in epithelial cells and that cuproptosis may interfere with tumor progression.

### CD74 is a key regulator in the progression of LUAD

**Fig. 3:**
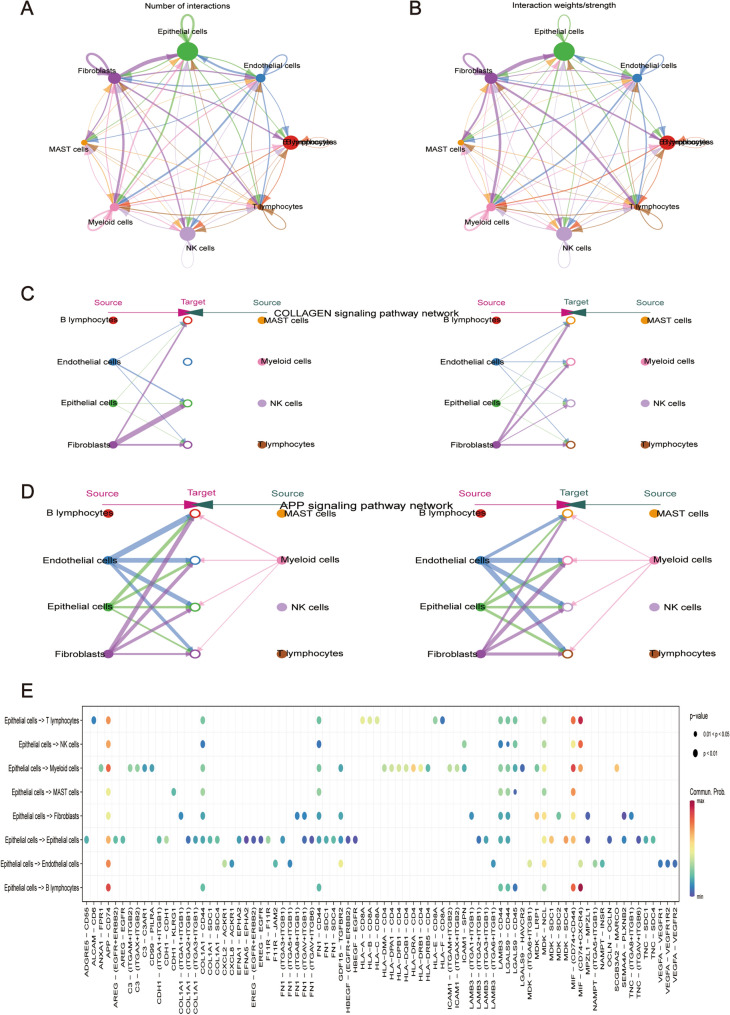
Cell Communication Analysis. **A** Network diagram illustrating the number of interactions between different cell subsets. **B** Network plot indicating the interaction strength among different cell subsets. Network diagrams presenting **C** Collagen and **D** Amyloid Precursor Protein (APP) related signaling pathways. **E** Bubble plots showing ligand-receptor pairs for each cell subset interacting with epithelial cells

Cell-cell interactions in LUAD multicellular systems were investigated by studying the expression of various ligand-receptor pairs. In LUAD tissues, we examined the interactions between various cell subsets. Figure 3A shows the number of interactions between cell subsets and Fig. 3B shows the weight of these interactions. We found that the collagen and APP signaling pathway networks accounted for the largest proportion of cell-cell communication. We then explored the interaction of these two pathways in cell communication (Fig. 3B, C). We found that epithelial cells did not interact with other cells through the collagen signaling pathway network. However, regarding the APP signaling pathway network, there was a significant interaction between epithelial cells and other cell subsets. Subsequently, we predicted ligand-receptor interactions between epithelial cell subsets and other cell subsets. We found that epithelial cells communicated with other cells via APP-CD74, MIF-(CD74 + CD44), and MIF-(CD74 + CXCR4) (Fig. 3D). Overall, CD74 seems to play a key role in the crosstalk between LUAD cells and promote the occurrence of tumor diseases.

### Construction of a prognostic model

**Fig. 4:**
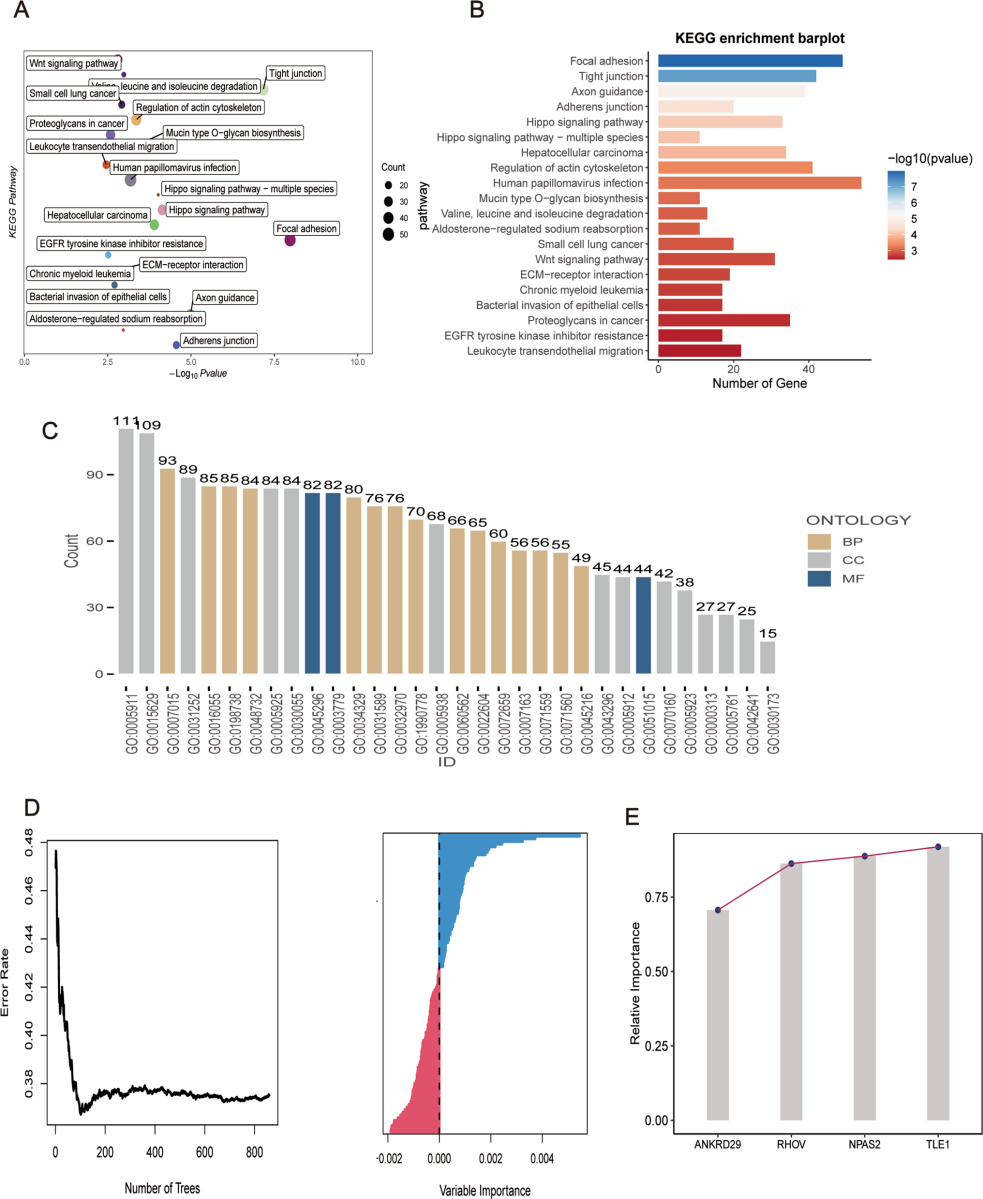
Differentially Expressed Gene-Enriched Pathway Analysis. **A**, **B** Bubble distribution plots and histograms illustrating Kyoto Encyclopedia of Genes and Genomes (KEGG) pathway enrichment of differentially expressed genes between epithelial cell subgroups and other subgroups. **C** Histogram presenting Gene Ontology (GO)-enriched pathways for expressed genes. **D** Graph showing the impact of the number of selected trees on the error rate, with the x-axis representing the number of decision trees and the y-axis representing the error rate. **E** Random forest classifier results based on Gini coefficient, with the x-axis representing genes and the y-axis representing importance indices

Because cuproptosis pathway activity is enriched in epithelial cell subgroups, we searched for DEGs among epithelial cell subgroups and other subgroups. After identifying these DEGs, clusterProfiler was used to functionally annotate them. KEGG pathway enrichment analysis showed that these genes were related to a variety of pathways, mainly, focal adhesion, tight junctions, and the Hippo signaling pathway (Fig. 4A, B). GO analysis showed that DEGs were mainly related to GO terms GO:0005911 and GO:0015629 (Fig. 4C).

Subsequently, univariate COX survival analysis was performed on DEGs using the tinyarray package (p < 0.01), and 112 genes were selected (Fig. 4D). Then, 4 prognostic genes, ANKRD29, RHOV, TLE1, and NPAS2, were selected from the 112 genes in the TCGA-LUAD cohort according to the random forest analysis results, and a prognostic model was constructed using them (Fig. 4E).

Riskscore = 0.0595*NPAS2 + 0.1717*TLE1 + 0.1217*RHOV + (-0.073)*ANKRD29.

**Fig. 5:**
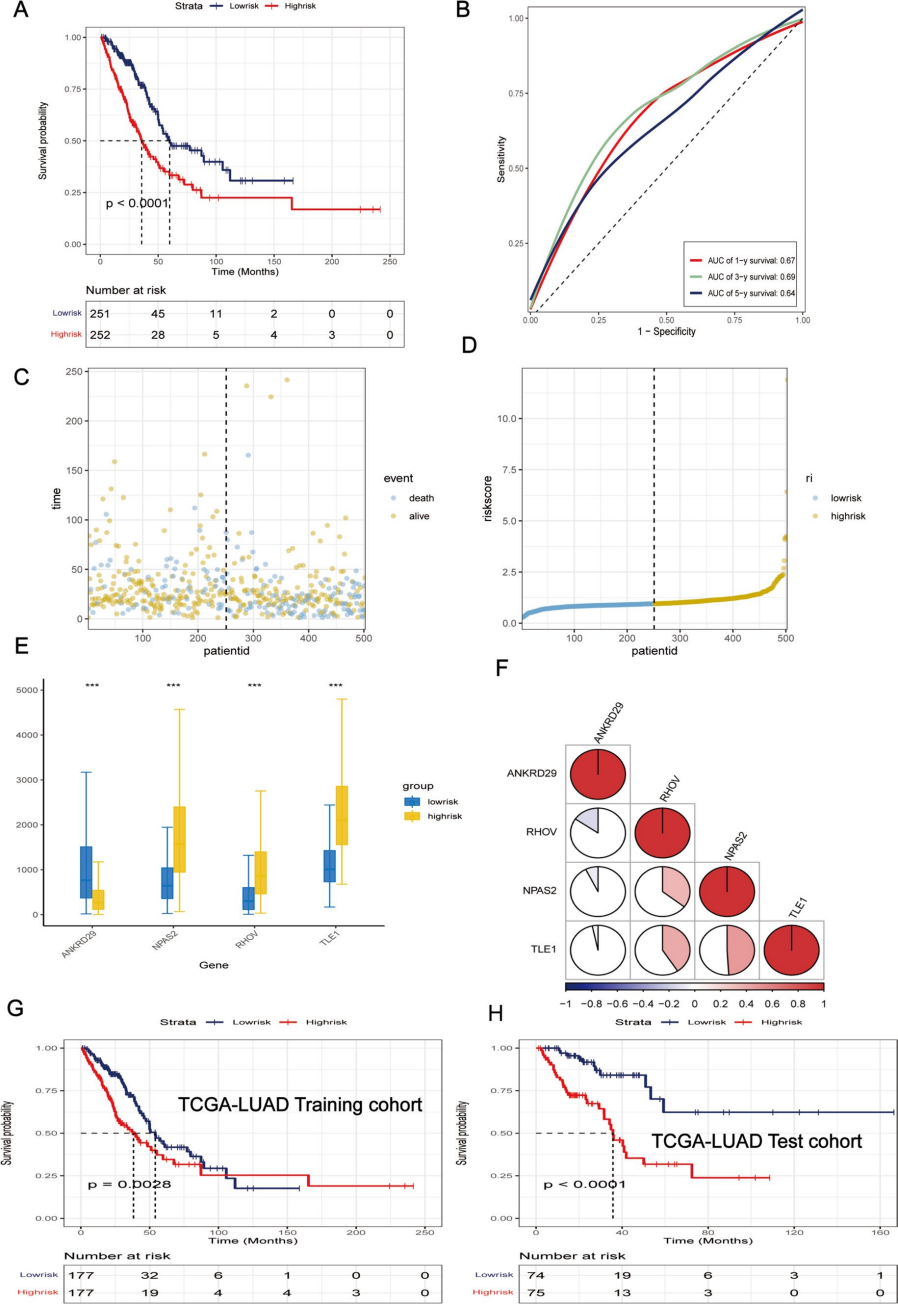
Prognostic Model Construction. **A** Kaplan-Meier (KM) survival curves for high- and low-risk patients in the Cancer Genome Atlas (TCGA) cohort. **B** Receiver Operating Characteristic (ROC) curves predicting 1-, 3-, and 5-year survival in the TCGA cohort. **C**, **D** Scatterplot depicting the survival distribution of high- and low-risk patients in the TCGA cohort. **E** Box plots showing differences in gene expression between high- and low-risk groups. **F** Pie chart presenting the correlation between modeled genes. **G**, **H** KM survival curves for high- and low-risk patients in the TCGA training and test sets, respectively

A K-M analysis showed that patients in the low-risk group had longer clinical survival than those in the high-risk group (Fig. 5A). The area under the ROC curve showed 1-, 3-, and 5-year survival rate values of 0.67, 0.69, and 0.64, respectively (Fig. 5B). Scatter plots show risk values and survival states(Figure 5 C, D). Besides, the box plot showed that NPAS2, RHOV, and TLE1 were highly expressed in the high-risk group, whereas ANKRD29 was highly expressed in the low-risk group (Fig. 5E). The relationships between these 4 genes are shown in Fig. 5F; TLE1 had the highest correlation with NPAS2. In addition, we divided TCGA-LUAD into training and validation cohorts (7:3), and the K-M results were consistent with the above results (Fig. 5G, H).

### TME assessment

Two methods were used to calculate the immune infiltration score: xCell and ssGSEA. The results were visualized using stacking diagrams, correlation heat maps, and scatter diagrams.

**Fig. 6:**
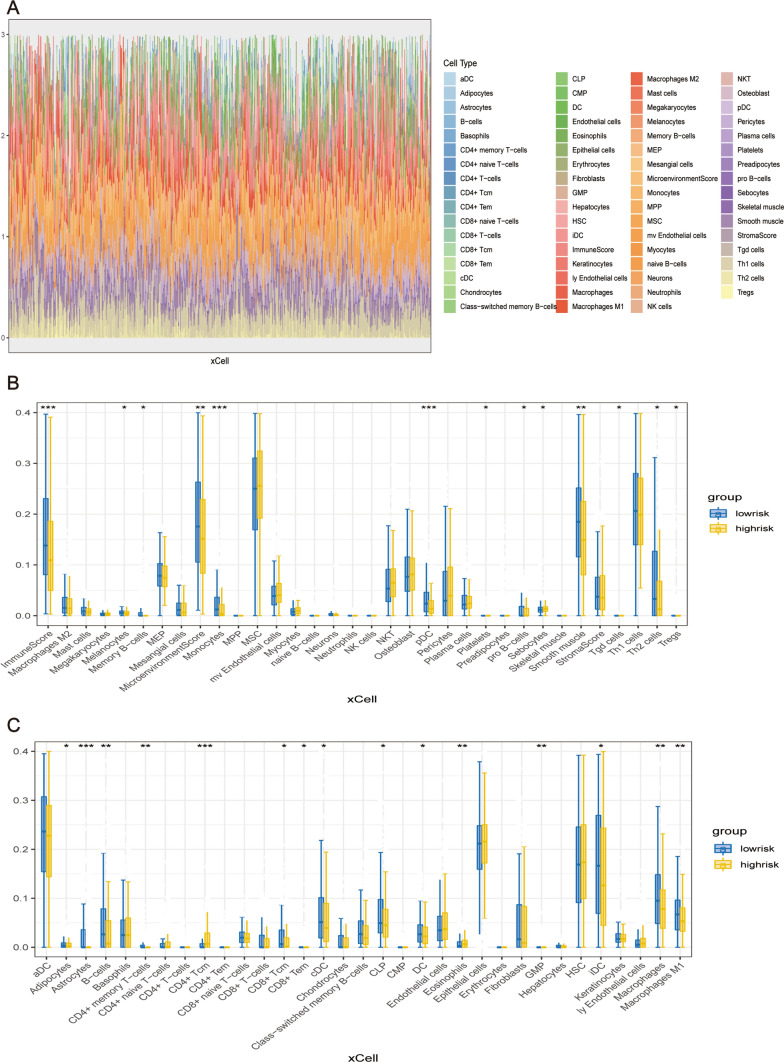
Immune Infiltration Analysis. **A** Heatmap illustrating variable percentages of immune cells among patients. **B**, **C** Box plots presenting differences in immune cell infiltration between high- and low-risk groups

Regarding the results from the xCell algorithm, the stack diagram showed the infiltration landscape of immune cell subsets in each patient; it could be seen that there were differences in the proportion of infiltrated immune cell subsets among the patients (Fig. 6A). The boxplot further showed that immune cell infiltration levels, such as pDC, Th2 cells, B cells, and M1 macrophages levels, were generally higher in low-risk patients than in high-risk patients (Fig. 6B, C). The overall ImmuneScore was also higher for the low-risk group of patients.

**Fig. 7:**
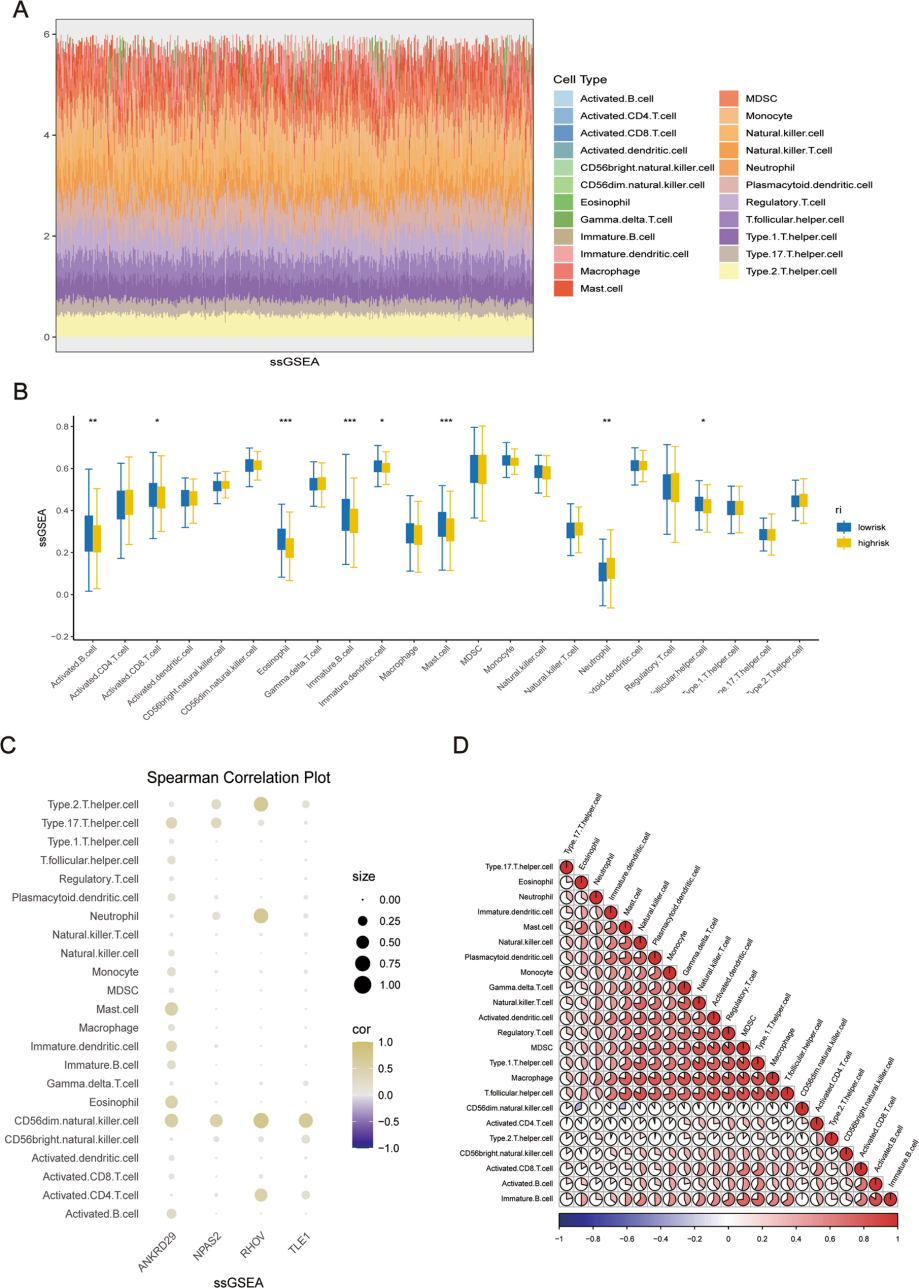
Single-Sample Gene Set Enrichment Analysis (ssGSEA). **A** Heatmap showing immune-related cell content in each patient as determined by ssGSEA. **B** Box plots presenting differences in immune cell content between high- and low-risk groups. **C** Bubble plots showing the expression of modeled genes in each immune cell. **D** Pie chart presenting the correlation between each immune-related cell

The stack plot and boxplot from the analysis with ssGSEA were consistent with the above results, and immune cell infiltration levels were generally higher in the low-risk group than in the high-risk group (Fig. 7A, B). In addition, Spearman’s correlation analyses showed a correlation between the genes and immune cells. TLE1 was identified as the most important gene in the model; therefore, we further focused on this gene.

TLE1 was positively correlated with CD56dim natural killer cells. In addition, CD56dim natural killer cells were associated with all prognosis-related genes in the model (Fig. 7C). Figure 7D shows the correlation between these immune cells. The higher the proportion of red, the higher the correlation between cell subgroups.

### Drug sensitivity prediction

**Fig. 8:**
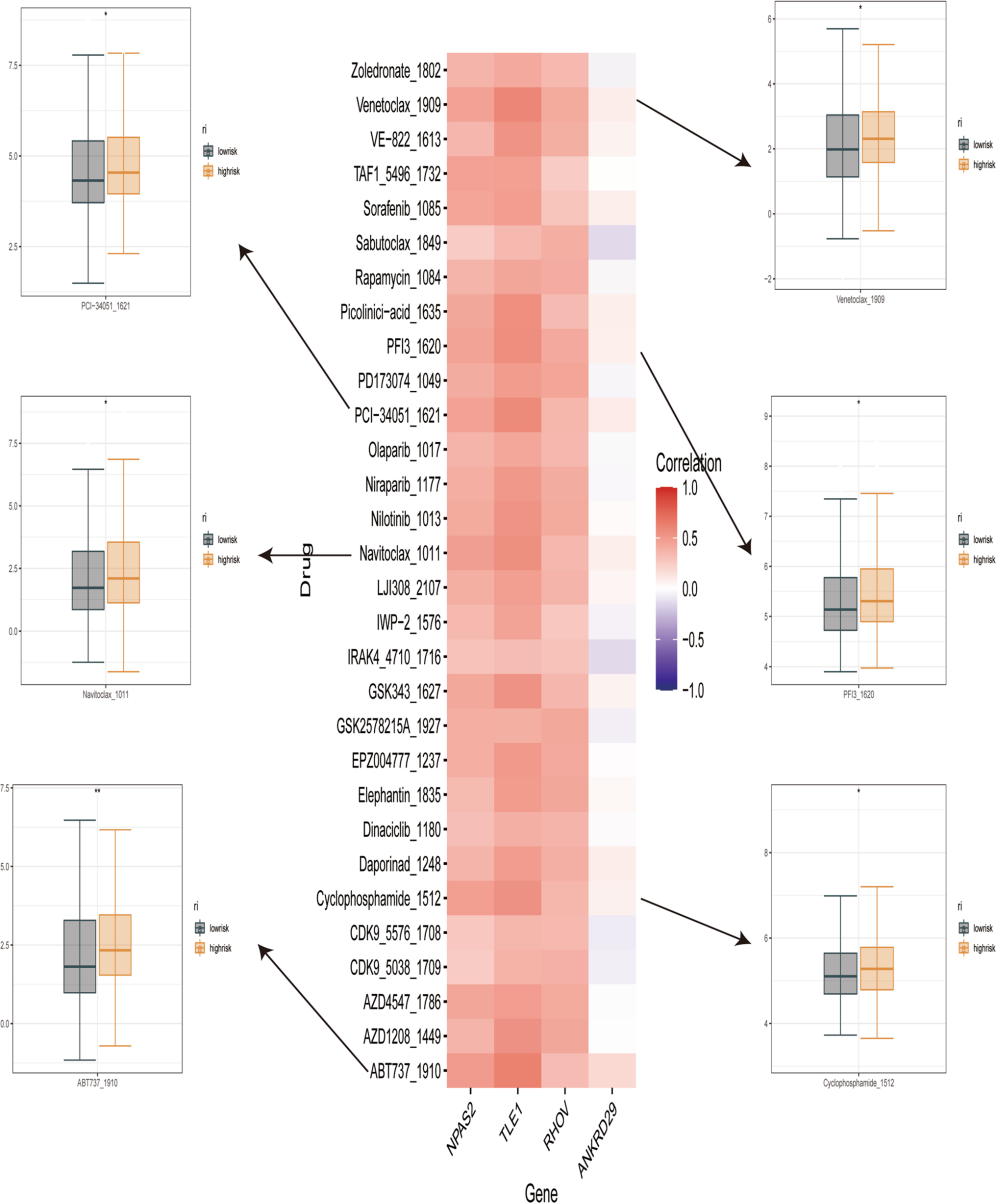
Drug Sensitivity Analysis. The heatmap displays the potential drug sensitivity associated with the modeled genes, while the box plot presents the half-maximal inhibitory concentration (IC50) values for patients in the high- and low-risk groups for the corresponding drug

The sensitivity score for drugs in the GDSC database was calculated using oncoPredict. Figure 8 shows the correlation between drug sensitiveness and prognostic genes. Predictions indicated that PCI-34,051 _ 1621, Navitoclax _1011, ABT737 _1910, Venetoclax _1909, PFI3 _ 1620, and Cyclophosphamide _1512 were the most suitable drugs for patients in the low-risk group.

#### In vitro experimental verification

**Fig. 9:**
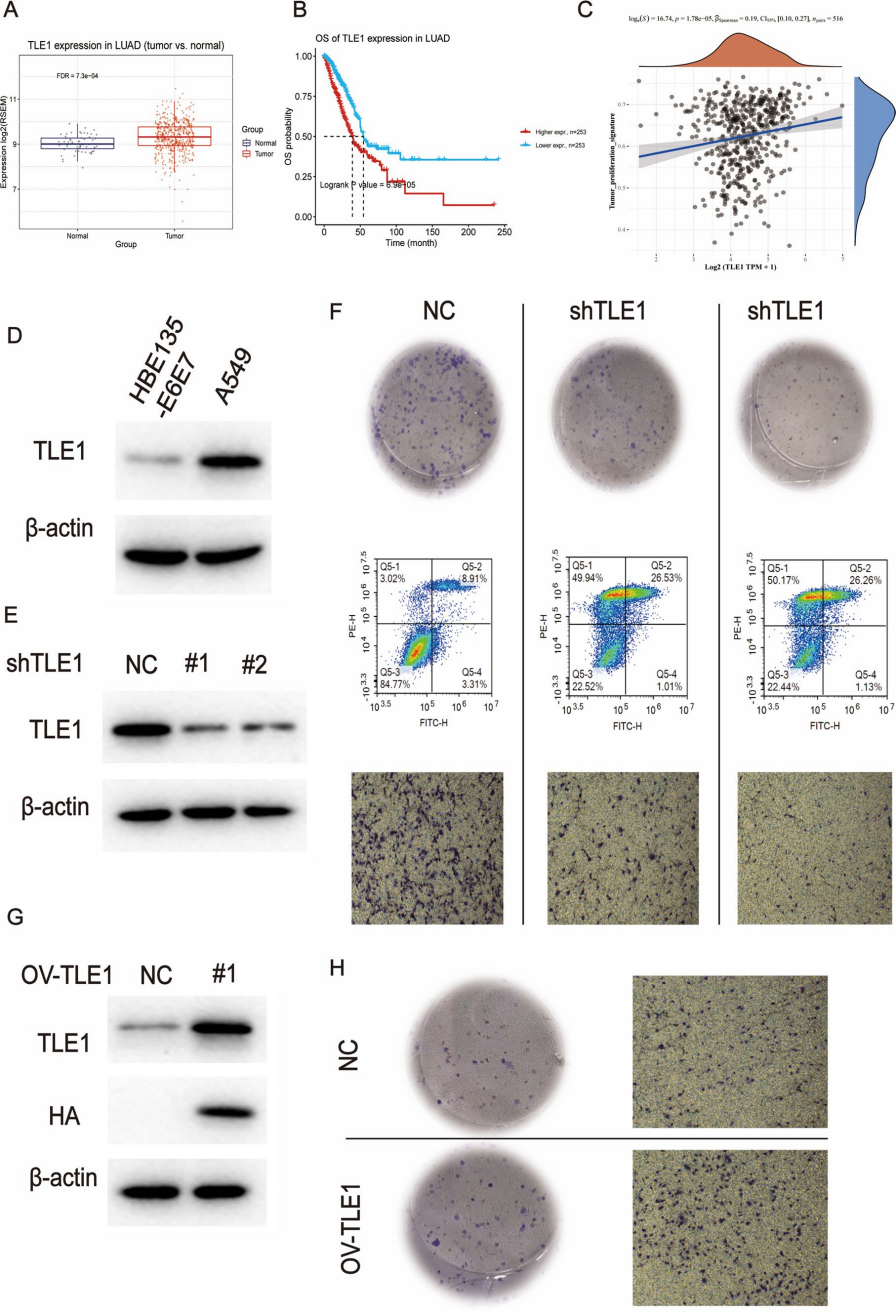
To verify the role of TLE1 in lung adenocarcinoma. **A** Box plots showed the expression of TLE1 in normal and tumor tissues. **B** Survival curves showed survival of patients with high and low expression of TLE1. **C** Scatter plots showed the expression of TLE1 associated with cell cycle. **D** Western blot showing expression of TLE1 in HBE135-E6E7 and A549 cells. **E** Western blot showing TLE1 knockdown efficiency in A549 cells by independent siRNA. **F** Colony formation was measured in cells transfected with control or si-TLE1. Transwell assay showed cell transfer in control cells compared to TLE1 knockdown cells. **G** TLE1 overexpression efficiency in NCI-H2073 cell line was verified by Western blot. **H** Colony formation was measured in cells transfected with control or overexpressing TLE1. Transwell assay showing cell transfer in control cells compared to TLE1 overexpressing cells

According to the public database, TLE1 transcription was significantly increased in LUAD tissues compared to that in normal lung tissues (Fig. 9A). Moreover, its abnormal expression indicated shorter overall survival (Fig. 9B), suggesting that TLE1 may be an important marker for determining the prognosis of patients with lung cancer. To further explore the potential mechanism by which a high expression of TLE1 may predict poor prognosis, we conducted pathway correlation analysis and found that TLE1 levels were positively correlated with the activation of tumor proliferation signaling, suggesting that an abnormal expression of TLE1 may activate rapid cycle transition mechanisms and endow lung cancer cells with antiapoptotic ability, thereby promoting the malignant proliferation of lung cancer cells (Fig. 9C). To further validate the correlation analysis, we determined the expression of TLE1 in normal bronchial epithelial cells and lung cancer cell lines. We found that A549 non-small cell lung cancer cells were significantly more likely to express TLE1 than normal bronchial epithelial cells (Fig. 9D).

In order to verify the role of TLE1 in lung cancer progression, on the one hand, we used two different shRNAs to knock down TLE1 in A549 cells that had a relatively high expression of this gene. The efficiency of the TLE1 knockdown was verified by western blot analysis (Fig. 9E). TLE1 knockdown significantly inhibited the clone formation ability of lung cancer cells and promoted apoptosis. In addition, TLE1 knockdown significantly inhibited the metastatic potential of lung cancer cells in vitro (Fig. 9F). On the other hand, we overexpressed TLE1 in NCI-H2073 cells that had a low expression of this gene, and verified the overexpression efficiency by western blot analysis (Fig. 9G). We found that TLE1 overexpression significantly promoted lung cancer cell clone formation and in vitro metastatic potential (Fig. 9H).

## Discussion

Lung cancer is the most common malignant tumor found in China, and its mortality rate is among the highest worldwide [1]. Patients with LUAD have a poor prognosis. In the current state of lung cancer screening, low-dose computed tomography screening can reduce mortality, but it is associated with a high false-positive rate [24, 25]. To prevent and control LUAD, it is crucial to determine reliable lung cancer risk characteristics and accurately predict the prognosis and survival of the patients [26]. Recent discoveries have led to the discovery of a new form of cell death called cuproptosis [19]. This copper-dependent cell death is caused by the direct binding of copper to acylated lipid components of the TCA cycle in mitochondrial respiration, leading to aggregation of acylated proteins, followed by reduction of iron-sulfur proteins, proteotoxicity, and, ultimately, cell death [19, 27–29]. In an attempt to induce cuproptosis, the small molecule anticancer drug elesclomol, which is a copper ionophore, has undergone clinical trials, but the results have not been satisfactory [30, 31]. Consequently, further research on cuproptosis and its regulatory role in LUAD is needed. Here, we comprehensively described the clinical significance of cuproptosis patterns in LUAD as well as their relationship with TME features. Furthermore, a cuproptosis scoring system was proposed to help doctors develop more effective immunotherapy strategies by improving the understanding of the TME.

In our investigation, we analyzed data from 503 patients with lung cancer (the TCGA-LUAD cohort) and 11 LUAD single-cell datasets from GSE131907. Based on cell characteristic markers, the cells were divided into eight cell subsets. The activity of the cuproptosis pathway was higher in the epithelial cell subsets, and carcinogenic pathways such as MAPK and TGF-β were enriched in this cell subset, revealing that tumors may originate from epithelial cells and that cuproptosis plays a crucial role in the pathogenesis of the disease. Subsequent cell communication analyses revealed that CD74 is an active element in the interaction between epithelial cell subsets and other cells. Fernandez-Cuesta et al. analyzed LUAD tissues from 25 non-smokers and identified a new somatic gene fusion, CD74-NRG1, that causes the EGF-like domain of NRG1 III-β3 to be expressed extracellularly, thereby, providing ligands for the ERBB2-ERBB3 receptor complex [32]. Xu et al. also proposed that CD74 is involved in the development of various types of cancer and demonstrated that CD74 can be used as a biomarker for predicting prognosis in patients with glioma [33]. These results suggest that CD74 is an important factor involved in the interaction between epithelial cells and other cells [34]. In addition, CD74 promotes the progression of LUAD [35].

Subsequently, we identified DEGs among epithelial cell and other cell subsets. We developed two clusters based on these DEGs and found a significant difference between the two groups in terms of prognosis, immune infiltration, and immune function. Because the response to clinical therapy may be adversely affected by the genomic alterations associated with LUAD, a random forest survival algorithm was used to establish the risk score. Using transcriptome sequencing, the risk score of patients with LUAD can be determined from pathological samples. A high-risk score was associated with shorter overall survival time, indicating adverse outcomes. In addition, the results of the immune infiltration analysis suggested that the clinical outcome of patients with cancer was significantly correlated with immune cell infiltration, indicating that cuproptosis may be related to tumor development and the TME.

Due to its significance in the prognostic model, we conducted experimental validation on Transducin-Like Enhancer of Split 1 (TLE1). TLE1 functions as a transcriptional corepressor with diverse cellular roles [36]. In the context of LUAD, TLE1 transcription was markedly elevated compared to normal lung tissues, and its abnormal expression correlated with reduced overall survival. Pathway correlation analysis further revealed a positive association between high TLE1 levels and the activation of tumor proliferation signaling, thereby promoting the malignant proliferation of lung cancer cells. Through knockdown experiments in A549 cells, we observed inhibited clonogenesis, induced apoptosis, and decreased metastatic potential upon TLE1 knockdown. Conversely, TLE1 overexpression in NCI-H2073 cells enhanced clonogenesis and metastatic potential. These findings highlight the pivotal involvement of TLE1 in LUAD progression and its potential as a prognostic marker.

There are still some shortcomings to this research. First, we analyzed public database data secondary to our analysis. Because of retrospective data selection bias, the results of the analysis are not as accurate as they should be. Well-designed prospective studies are required to validate our findings. In addition, there was a lack of in vivo experiments for further verification, and the mechanism by which TLE1 regulates the phenotypic alterations of LUAD cells needs to be elucidated.

## Supplementary Information

Supplementary material 1

## Data Availability

The original contributions presented in the study are included in the article/supplementary material, further inquiries can be directed to the corresponding author.

## References

[1] Ferlay J, Colombet M, Soerjomataram I, Parkin DM, Piñeros M, Znaor A (2020). Cancer statistics for the year, an overview. Int J Cancer.

[2] Brunelli A, Charloux A, Bolliger CT, Rocco G, Sculier JP, Varela G (2009). ERS/ESTS clinical guidelines on fitness for radical therapy in lung cancer patients (surgery and chemo-radiotherapy). Eur Respir J.

[3] Spella M, Stathopoulos GT (2021). Immune resistance in lung adenocarcinoma. Cancers.

[4] Antanavičiūtė I, Rysevaitė K, Liutkevičius V, Marandykina A, Rimkutė L, Sveikatienė R (2014). Long-distance communication between laryngeal carcinoma cells. PLoS ONE.

[5] Liang JY, Wang DS, Lin HC, Chen XX, Yang H, Zheng Y (2020). A Novel ferroptosis-related gene signature for overall survival prediction in patients with Hepatocellular Carcinoma. Int J Biol Sci.

[6] Yu SL, Chen HY, Chang GC, Chen CY, Chen HW, Singh S (2008). MicroRNA signature predicts survival and relapse in lung cancer. Cancer Cell.

[7] Chen CZ, Chen JZ, Li DR, Lin ZX, Zhou MZ, Li DS (2013). Long-term outcomes and prognostic factors for patients with esophageal cancer following radiotherapy. World J Gastroenterol.

[8] Li J, Zhou Z, Dong J, Fu Y, Li Y, Luan Z (2021). Predicting breast cancer 5-year survival using machine learning: a systematic review. PLoS ONE.

[9] Gao W, Wang X, Zhou Y, Wang X, Yu Y (2022). Autophagy, ferroptosis, pyroptosis, and necroptosis in tumor immunotherapy. Signal Transduct Target Ther.

[10] Dong T, Liao D, Liu X, Lei X (2015). Using small molecules to dissect non-apoptotic programmed cell death: necroptosis, ferroptosis, and pyroptosis. ChemBioChem.

[11] Tang R, Xu J, Zhang B, Liu J, Liang C, Hua J (2020). Ferroptosis, necroptosis, and pyroptosis in anticancer immunity. J Hematol Oncol.

[12] Zhou B, Liu J, Kang R, Klionsky DJ, Kroemer G, Tang D (2020). Ferroptosis is a type of autophagy-dependent cell death. Semin Cancer Biol.

[13] Dixon SJ, Lemberg KM, Lamprecht MR, Skouta R, Zaitsev EM, Gleason CE (2012). Ferroptosis: an iron-dependent form of nonapoptotic cell death. Cell.

[14] Cobbett C (2003). Heavy metals and plants - model systems and hyperaccumulators. New Phytol.

[15] Sen S, Won M, Levine MS, Noh Y, Sedgwick AC, Kim JS (2022). Metal-based anticancer agents as immunogenic cell death inducers: the past, present, and future. Chem Soc Rev.

[16] Carter KP, Young AM, Palmer AE (2014). Fluorescent sensors for measuring metal ions in living systems. Chem Rev.

[17] Uauy R, Olivares M, Gonzalez M (1998). Essentiality of copper in humans. Am J Clin Nutr.

[18] Linder MC, Hazegh-Azam M (1996). Copper biochemistry and molecular biology. Am J Clin Nutr.

[19] Tsvetkov P, Coy S, Petrova B, Dreishpoon M, Verma A, Abdusamad M (2022). Copper induces cell death by targeting lipoylated TCA cycle proteins. Science.

[20] Li H, Yu L, Zhang X, Shang J, Duan X (2022). Exploring the molecular mechanisms and shared gene signatures between rheumatoid arthritis and diffuse large B cell lymphoma. Front Immunol.

[21] Li H, Zhang X, Shang J, Feng X, Yu L, Fan J (2023). Identification of NETs-related biomarkers and molecular clusters in systemic lupus erythematosus. Front Immunol.

[22] Aibar S, González-Blas CB, Moerman T, Huynh-Thu VA, Imrichova H, Hulselmans G (2017). SCENIC: single-cell regulatory network inference and clustering. Nat Methods.

[23] Wang H, Yang F, Luo Z (2016). An experimental study of the intrinsic stability of random forest variable importance measures. BMC Bioinformatics.

[24] Aberle DR, Adams AM, Berg CD, Black WC, Clapp JD, Fagerstrom RM (2011). Reduced lung-cancer mortality with low-dose computed tomographic screening. N Engl J Med.

[25] Hasan N, Kumar R, Kavuru MS (2014). Lung cancer screening beyond low-dose computed tomography: the role of novel biomarkers. Lung.

[26] Li Y, Ge D, Gu J, Xu F, Zhu Q, Lu C (2019). A large cohort study identifying a novel prognosis prediction model for lung adenocarcinoma through machine learning strategies. BMC Cancer.

[27] Li SR, Bu LL, Cai L (2022). Cuproptosis: lipoylated TCA cycle proteins-mediated novel cell death pathway. Signal Transduct Target Ther.

[28] Tang D, Chen X, Kroemer G (2022). Cuproptosis: a copper-triggered modality of mitochondrial cell death. Cell Res.

[29] Wang Y, Zhang L, Zhou F (2022). Cuproptosis: a new form of programmed cell death. Cell Mol Immunol.

[30] Zhang J, Duan D, Song ZL, Liu T, Hou Y, Fang J (2021). Small molecules regulating reactive oxygen species homeostasis for cancer therapy. Med Res Rev.

[31] Hasinoff BB, Wu X, Yadav AA, Patel D, Zhang H, Wang DS (2015). Cellular mechanisms of the cytotoxicity of the anticancer drug elesclomol and its complex with Cu(II). Biochem Pharmacol.

[32] Fernandez-Cuesta L, Plenker D, Osada H, Sun R, Menon R, Leenders F (2014). CD74-NRG1 fusions in lung adenocarcinoma. Cancer Discov.

[33] Xu S, Li X, Tang L, Liu Z, Yang K, Cheng Q (2021). CD74 correlated with Malignancies and Immune Microenvironment in Gliomas. Front Mol Biosci.

[34] McClelland M, Zhao L, Carskadon S, Arenberg D (2009). Expression of CD74, the receptor for macrophage migration inhibitory factor, in non-small cell lung cancer. Am J Pathol.

[35] Kindt N, Lechien JR, Nonclercq D, Laurent G, Saussez S (2014). Involvement of CD74 in head and neck squamous cell carcinomas. J Cancer Res Clin Oncol.

[36] Yuan D, Yang X, Yuan Z, Zhao Y, Guo J (2017). TLE1 function and therapeutic potential in cancer. Oncotarget.

